# The idea called India

**DOI:** 10.4103/0970-0358.41131

**Published:** 2008

**Authors:** The Lancer

Dear Sir,

The last issue of IJPS (Vol. 40, 2007 December) is quite remarkable for its content. The lead article is written by Kalpesh Gajiwala, who was not only entirely trained in India but has now written on the difficult subject of speech pathology, with Indian languages in mind and the classical Sanskrit alphabet as their foundation. To many of my generation who had to go via the English language to understand speech pathology but who had sensed that Sanskrit had a very logical arrangement of the expressed sound this attempt is not only heartening but also has a touch of the audacious. Lest my commentary be considered parochial it is worth remembering that Panini, the great Sanskrit grammarian, according to today’s political geography would be considered an Afghan national.

The issue also has touching obituaries of two very worthy members of our fraternity – Noshir Antia and Manohar Keswani. While Antia’s forefathers escaped to India from Iran to avoid religious persecution, Keswani was uprooted from his native Sind when the Indian subcontinent was partitioned. India thus was their somewhat new home. Yet both developed new ideas which were fashioned by modern science but were specifically useful to their new country. Kalpesh Gajiwala stands on the shoulders of these stalwarts who prospered not in a political entity called India but a liberal idea called India.

In the generation when Antia and Keswani were active many a unit in the country had to take shelter in journals abroad to enable them to express themselves. The British Journal of Plastic Surgery did this with considerable grace. That journal has now morphed into an international journal and the IJPS too now has considerable presence of those who do not practise in India. This must never be viewed as any sort of Indian clout. In fact both journals must be viewed as part of a global scientific knowledge pool.

Patanjali (probably a contemporary of Panini), the other great Indian thinker who gave us the Yogic school of philosophy classified minds into: indolent, leading to wretchedness; feverish, leading to turmoil and strife and engrossed and later focused which helped foster analytical ability and creativity. The role of editors through millennia has been to discover and nurture such minds dispassionately.

For example,

In their hands (the editors')

The clever become wise

Dry truth, with wisdom ties

Knowledge grows and thrives

The current editorial team of IJPS must be complimented for having brought the journal to its present state. The task ahead is not easy because the higher you get the easier you are likely to slip and fall. Be that as it may, by a coincidence, the article by Gajiwala in this issue is reviewed by ACH Watson, a very discerning and dispassionate past editor of the then British Journal of Plastic Surgery, and the current editor of IJPS was his trainee in the late eighties. This is the Guru-Shishya parampara (the Teacher and Student tradition) come to fruit across oceans.

